# The effect of marital status on stage at diagnosis and survival in Saudis diagnosed with colorectal cancer: cancer registry analysis

**DOI:** 10.1038/s41598-021-88042-9

**Published:** 2021-04-21

**Authors:** Mesnad Alyabsi, Majed Ramadan, Mohammed Algarni, Kanan Alshammari, Abdul Rahman Jazieh

**Affiliations:** 1 Population Health Research Section, King Abdullah International Medical Research Center, P.O. Box 22490, Riyadh, 11426 Saudi Arabia; 2 Population Health Research Section, King Abdullah International Medical Research Center, King Abdul Aziz Medical City, Jeddah, 22384 Saudi Arabia; 3 Oncology Department, Ministry of National Guard - Health Affairs, Riyadh, Saudi Arabia; 4 King Saud Bin Abdulaziz University for Health Sciences, P.O. Box 22490, Riyadh, 11426 Saudi Arabia; 5 King Abdullah International Medical Research Center, P.O. Box 22490, Riyadh, 11426 Saudi Arabia

**Keywords:** Colon cancer, Rectal cancer

## Abstract

Colorectal cancer (CRC) is the most common cancer in males and third in females in Saudi Arabia, with the majority (66%) diagnosed at a late stage. We evaluated the effect of marital status on stage at diagnosis and CRC survival. We hypothesized that married patients would be more likely to present at an early stage and have higher survival than unmarried patients. The Ministry of National Guard-Health Affairs (MNG-HA) cancer registry was used to identify patients diagnosed with CRC from 2009 to 2017. A competing risk analysis was performed to assess the 5-year CRC-specific survival, adjusting for potential confounders. The Kaplan–Meier method and the Cox regressions were used to assess survival. Two-thirds (76.50%) of the 936 CRC patients were married, 11.64% were unmarried, and 11.86% had an unknown marital status. With multiple imputation-based analysis, the multivariate analysis indicated that unmarried patients were 52% more likely to present at an advanced stage [adjusted odds ratio (aOR) 1.52; 95% CI 1.33–1.73], and had a 30% higher risk of death due to CRC compared to the married patients (aHR 1.30; CI 1.17, 1.44). Future CRC screening and survivorship programs should assess the needs of the vulnerable unmarried population. Interventions supporting the early detection of CRC in this population may be beneficial in the long term and lead to improved cancer outcomes.

## Introduction

Colorectal cancer (CRC) remains the most prevalent cancer in Saudi males and the third in Saudi females. The 2016 estimated age-standardized incidence rates were 12.9 and 9.5 per 10^5^ in males and females^1^. Though the national estimates indicated that the 5-year overall survival remained the same from 1994 to 2000 (44.7%) and from 2001 to 2004 (44.6%)^2–4^, recent estimates for the elderly showed a small survival improvement (54%)^5^. However, this improvement is lower than the survival estimates reported internationally (66–68%)^6–8^.

The recent increase in CRC survival is attributed to several clinical and non-clinical factors^6,9^. In addition to the advancement in CRC treatment through robotic surgical interventions and systemic therapy, other clinical factors such as early-stage diagnosis through screening also contributed to the higher survival^10^. However, non-clinical factors such as the patients’ level of awareness regarding CRC and preventive measures, the level of communication with a provider, the distance to a healthcare facility, and social support are less understood. Though prior Saudi Arabian research indicated a poor level of awareness^11,12^, no previous study assessed the impact of the marital status on the stage at diagnosis and survival in the Saudi population.

Several international studies assessed the impact of marital status on CRC outcomes, with diverse findings^13–18^. Using the Surveillance, Epidemiology, and End Results (SEER) data, the majority of the studies reported that married patients had better survival than unmarried patients, whether the rate of surgery receipt is similar^16^ or not^13^. The survival advantage of married patients is proportionally associated with the stage at diagnosis, with the highest survival advantage in stage III CRC^16^. Nonetheless, in multivariate analyses, adjusted for age at diagnosis, the survival advantage tends to reduce^13^ or dissipate in rectal cancer patients with multivariate adjustment for demographic variables, stage, and treatment^18^. A Chinese study assessing CRC survival in surgically treated colon cancer patients reported that married patients had higher survival than unmarried patients, despite a similar stage of cancer and adjuvant therapy use^15^. Contrary to these studies, marital status was not an independent predictor of CRC survival in a tertiary hospital in the United States (US)^19^. The impact of marital status on CRC outcomes is still unclear.

Of the social and psychological factors that influence cancer outcomes, marital status is a significant predictor of morbidity and mortality^20–25^. Compared to married patients, unmarried patients are more likely to have a delayed diagnosis^20^, experience faster cancer progression and metastasis as a chronic stressor^24^, and slow recovery after surgery for patients with a low-quality marriage^26^. Marital status as social support is associated with reduced mortality in patients diagnosed with chronic diseases^22^. Notably, marital status is more predictive of cancer survival than chemotherapy^14^. Given the vital role that marital status plays as social support for CRC patients, the present study was designed to assess the influence of marital status on the CRC stage at diagnosis and survival in CRC patients. We hypothesize that married CRC patients will be more likely to be diagnosed at an early stage and have higher survival than unmarried patients. This study’s findings will support practitioners who attend to patients during the survivorship phase of the cancer continuum.

## Methods

### Study design and data source

The Institutional Review Board of King Abdullah International Medical Research Center (IRB#139 RC20/029/R) approved the study after informed consent was obtained from all subjects. The current study is a retrospective cohort, using data from the Cancer Registry of the Ministry of National Guard-Health Affairs (MNG-HA). The information in the cancer registry was verified with hospital records, diagnostic procedures, pathology reports, and death certificates. The registry records all cases diagnosed and treated at King Abdulaziz Medical City (KAMC) in Riyadh. The Institutional Review Board of King Abdullah International Medical Research Center approved this study (RC20/383/R). All extracted data were de-identified, and all methods were performed in accordance with the Declaration of Helsinki.

### Identification of patients

All individuals aged ≥ 18 years, diagnosed with a first primary invasive, malignant colorectal cancer (International Classification of Diseases: ICD-10 C18–C20) from 2009 to 2017, registered in the MNG-HA hospital system, and followed-up to December 31, 2017, were eligible for analysis.

### Covariates and outcome variables

Several demographic and clinical variables were retrieved for analysis. These included age at diagnosis, gender, tumor stage, grade, morphology, and the site as covariates in the adjusted models. Treatment information included chemotherapy status, surgery receipt, and radiotherapy status as covariates in the adjusted models. Marital status was gathered in four categories: married, single (never married), divorced, widowed, and unknown (missing). We combined the three unmarried groups (single, separated/divorced, or widowed) to obtain a larger number of unmarried patients. The stage variables included localized, regional, distant, and unknown stages. To assess the risk of an advanced stage at diagnosis, we combined regional and distant stages in a single category. Multiple imputation was used to provide a reliable estimate for the missing marital status values. Survival is recorded in the database as the number of months from the date of diagnosis to the date of death, the date of last alive, and the last follow-up date on December 31, 2017, whichever occurred first. The underlying cause of death, as mentioned on the hospital death notification, was used to indicate the patients’ death status.

### Multiple imputation

After the exclusion of cases based on the criteria described, marital status was the only variable with a high proportion (11%) of missing values (monotone missing pattern or missing at random (MAR). Multiple imputation was used to approximate marital status for unknown values (n = 111)^27,28^. We used the SAS Multiple Imputation procedure to generate 15 imputed data sets for marital status values with the logistic regression imputation method (PROC MI with LOGISTIC in the MONOTONE statement [SAS version 9.4; SAS Institute, Cary, NC])^29^. A logistic regression model was fitted for marital status by the maximum likelihood method. Under the MAR assumption, all variables were included to derive the imputation model^27,29^. Simulation studies comparing multiple imputation to complete-case analyses suggest that excluding observations with missing data can considerably bias regression coefficients. Such bias can be reduced via multiple imputation^28,30^.

### Statistical analysis

Descriptive statistics for the patient baseline characteristics by marital status frequencies were compared using the likelihood Chi-square and Fisher exact test with the Monte Carlo approach. Diagnosis at an advanced vs. localized stage by marital status was compared while controlling for the covariates. We obtained the odds ratios and 95% confidence intervals (CI) from the multivariate logistic regression models. Using the Kaplan–Meier method, we compared the death rate due to CRC between marital status (married vs. unmarried) and generated the survival curves. Survival estimates were compared using the log-rank test. The impact of marital status on the survival outcomes was analyzed using multivariate Cox proportional hazards regression models for colorectal cancer-specific mortality and reported over the 5-years. The assumption of proportional hazards was evaluated by examining the scaled Schoenfeld residuals. Based on previous studies, we adjusted for the demographic variables, biological factors, and treatment (gender, age at diagnosis, stage, grade, site, morphology, chemotherapy status, surgery, and radiotherapy status)^15,18,31^. The stepwise selection method with *P* < 0.15 as the criterion for entry and *P* > 0.05 as the criterion for removal was used to select covariates for the final multivariate models. Only significant predictors remained in the model except for the tumor site. The threshold of 0.05 was used to determine significance. Statistical analyses were performed using SAS version 9.4 (SAS Institute, Cary, NC).

### Ethics approval and consent to participate

King Abdullah International Medical Research Center approved this study (RC20/383/R).

## Results

**Figure 1:**
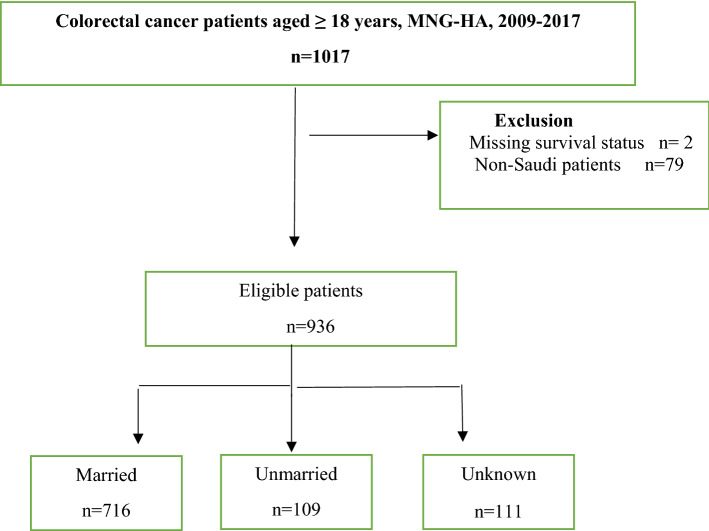
Eligibility criteria for the study population.

Of the 1017 eligible patients, 81 (8.1%) were excluded because the survival status was unknown or they were non-Saudi patients (Fig. 1). Most non-Saudi patients did not continue their treatment in Saudi Arabia and possibly decided to receive it in their home country. The majority were from diverse ethnicities and geographic locations (Supplementary Tables 1–3). We identified 936 eligible patients aged 18 years or older and diagnosed with CRC from 2009 to 2017. The patient demographic and clinical characteristics differed significantly between the marital status groups (Table 1). Of the sample, 716 (76.5%) were married at the time of diagnosis, and 109 patients (11.6%) were unmarried. The unmarried patients were more likely to be male and presented with worse tumor behavior, such as an advanced tumor stage (*P* < 0.0001). The unmarried patients (22%) were diagnosed at a younger age (18–39 years) compared to the married patients (5%, *P* < 0.0001). Just more than a third (35.8%) of the unmarried patients were diagnosed at an older age (70–98 years). About half of the unmarried patients (48.6%) and 41.2% of the married patients were diagnosed at a regional stage. The advanced stage (regional and distance) was highest in the unmarried patients (85.1%), compared to 73.9% in the married patients. The majority of the married (77.4%) and unmarried patients (71.6%) had moderately differentiated cancer grades. Most (84.5%) of the married patients had mucinous adenocarcinoma, compared to 78.9% of the unmarried patients. The treatment distribution pattern (chemotherapy, surgery, and radiotherapy) was somewhat similar for both groups.

**Table 1:** Demographic, clinical and pathological characteristics of saudi's colorectal cancer patients at MNG-HA (2009–2017)^a^.

Patients characteristics	Marital status (N^d^ = 936)			
	Married	Unmarried	Unknown	*P* value^b^
Total	n^e^ = 716	n = 109	n = 111	
	n (%)	n (%)	n (%)	
**Gender**				< 0.0001
Male	433 (60.5)	77 (70.6)	67 (60.4)	
Female	283 (39.5)	32 (29.4)	44 (39.6)	
**Age at diagnosis**				< 0.0001
10–39	36 (5.03)	23 (22)	10 (9)	
40–49	84 (11.7)	9 (8.3)	25 (22.5)	
50–59	192 (26.8)	18 (16.5)	30 (27)	
60–69	199 (27.8)	19 (17.4)	27 (24.3)	
70–98	205 (28.6)	40 (35.8)	19 (17.1)	
**Stage at diagnosis**				< 0.0001
Distant metastasis	234 (32.7)	35 (32.1)	43 (38.7)	
Regional	295 (41.2)	53 (48.6)	35 (31.5)	
Localized	165 (17.6)	16 (14.7)	27 (24.3)	
Other	22 (3.1)	5 (4.6)	35 (31.5)	
**Pathological grading**				< 0.0001
Well differentiated	20 (2.8)	5 (4.6)	4 (3.6)	
Moderately differentiated	554 (77.4)	78 (71.6)	87 (78.4)	
Poorly differentiated	50 (7)	6 (5.5)	7 (6.3)	
Others	92 (12.6)	20 (18.4)	13 (11.7)	
**Tumor morphology**				< 0.0001
Adenocarcinoma (AC), NOS	12 (1.7)	3 (2.8)	0	
Mucinous AC	605 (84.5)	86 (78.9)	99 (89.2)	
Mucin-producing AC	7 (1)	1 (1)	3 (2.7)	
Signet ring cell carcinoma	46 (6.4)	5 (4.6)	4 (2.6)	
AC in villous/tubuvillous adenoma	8 (1.1)	3 (2.8)	3 (2.7)	
Others^f^	38 (5.3)	11 (10.1)	2 (1.8)	
**Tumor site**				< 0.0001
Right colon	117 (16.3)	19 (17.5)	14 (12.6)	
Left colon	227 (31.8)	25 (23)	37 (36.1)	
Unspecified colon site	371 (51.9)	65 (59.7)	57 (51.4)	
**Chemotherapy**				0.0019
Yes	296 (41.3)	44 (40.37)	37 (33.3)	
No	420 (58.6)	65 (59.6)	74 (66.6)	
**Surgery**				0.0018
Yes	308 (43)	48 (44.04)	39 (35.14)	
No	408 (57)	61 (56)	72 (64.87)	
**Radiotherapy**				0.013
Yes	71 (9.92)	11 (10.1)	8 (7.2)	
No	645 (90.1)	98 (89.9)	103 (92.8)	

^a^Data represent Saudi patients registered in MNG-HA hospitals system between January 1 2009 and December 31 2017.

^b^*P* values refer to comparisons between marital status groups using Fisher exact test.

^c^*P* values refer to comparisons between marital status groups using Fisher exact test.

^d^"N" total sample size.

^e^"n" sample size for each group.

^f^Other categories for morphology with small number of subjects.

**Figure 2:**
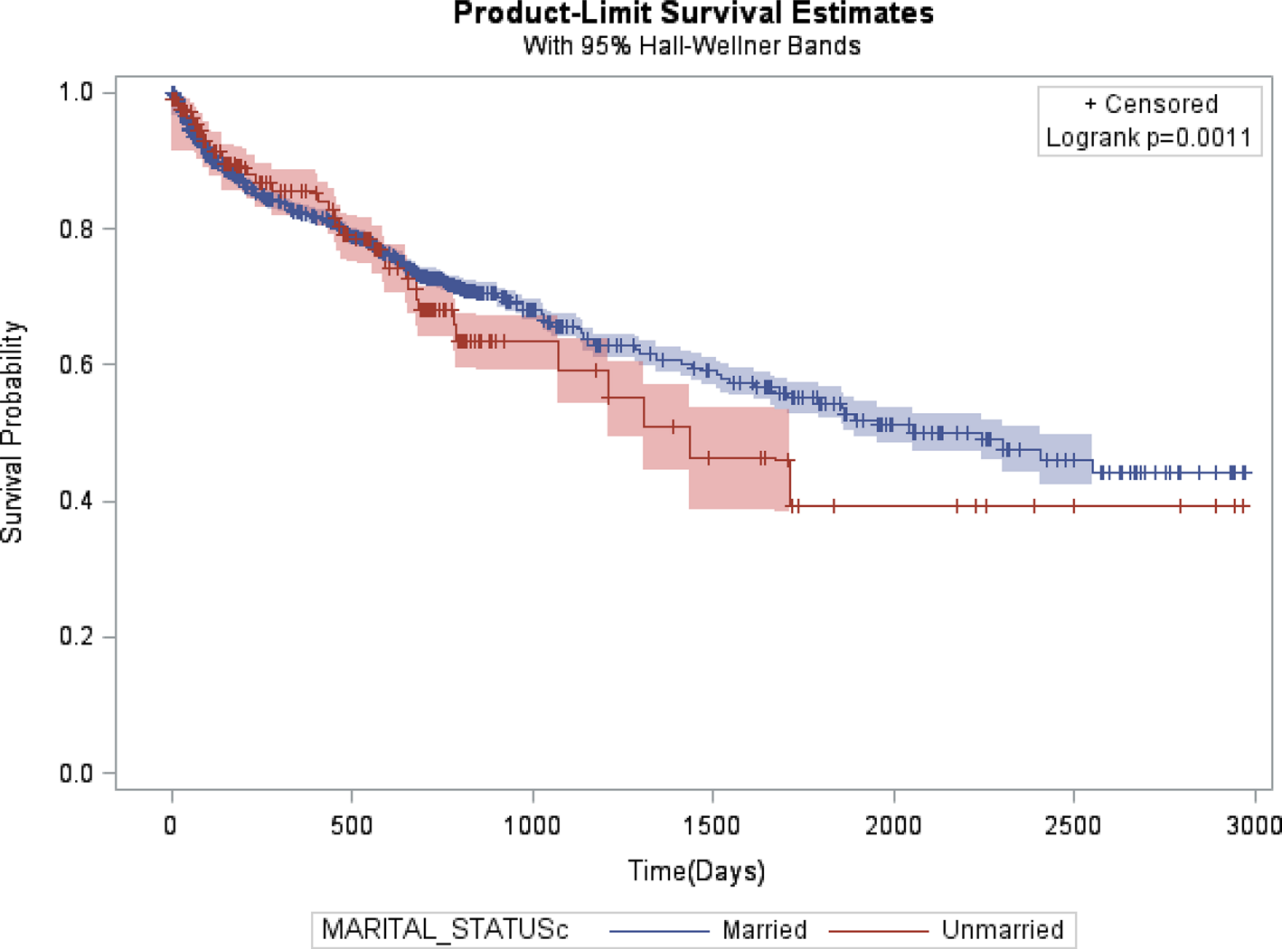
Kaplan–Meier cancer-specific survival curves comparing colorectal cancer by marital status.

**Figure 3:**
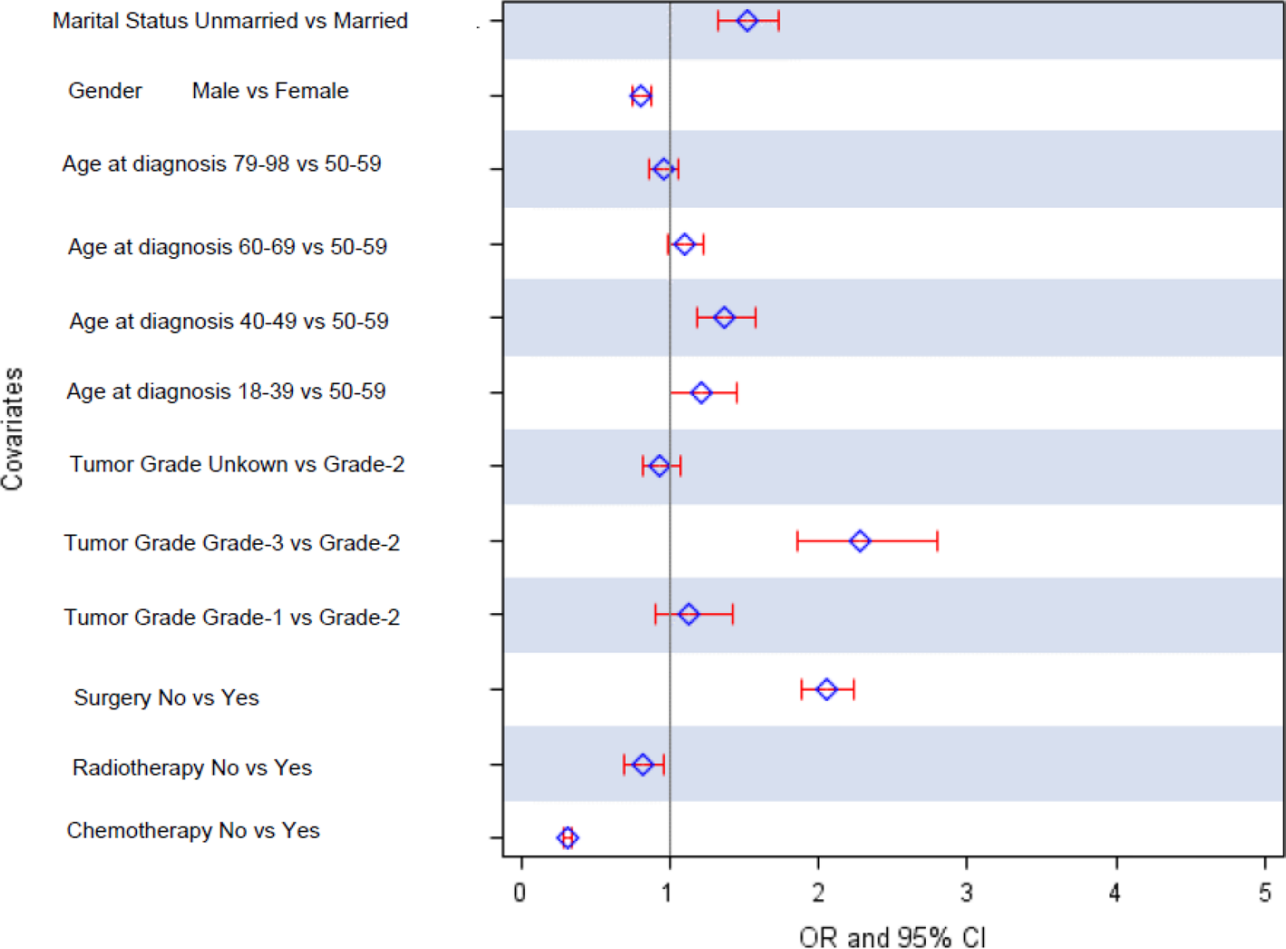
Association between marital status and stage at diagnosis in colorectal cancer patients.

The Kaplan–Meier survival curve was generated to compare the 5-year cancer-specific survival (Fig. 2). The married patients had a significantly higher 5-year cancer-specific survival than the unmarried patients. The log-rank tests indicated that the married patients had better survival outcomes than the unmarried patients (54% vs. 40% *P* < 0.0009). With the multivariate analysis (Table 2), overall, the married patients had a significant survival advantage over the unmarried patients after adjusting for demographic variables and clinical factors. The unmarried patients had a higher risk of death from CRC than the married patients (aHR 1.30; CI 1.17, 1.44). Other predictors, independently associated with an excess risk of death from CRC, were being female, older age at diagnosis (70–98 years), with a poorly differentiated grade, a mucinous adenocarcinoma histologic type, and no treatment (chemotherapy, surgery, or radiotherapy) (Table 2). We also examined the association between marital status and the stage at diagnosis after controlling for relevant patient characteristics (Table 3 and Fig. 3). We found that the unmarried CRC patients had significantly greater odds of being diagnosed at an advanced colorectal cancer stage than the married patients [adjusted odds ratio (aOR) 1.52; 95% CI 1.33–1.73]. In a supplementary analysis that assesses the impact of marital status on CRC risk of death stratified by gender, we found that unmarried women were 29% more likely to die compared to married women (HR) 1.29; 95% CI 1.15–1.44] but the reverse was true for men (Supplementary Table S4).

**Table 2:** Multivariate survival analysis for evaluating the influence of marital status on colorectal cancer over 5 years , MNG-HA, 2009–2017^a^.

	Complete-case analysis^b^		Multiple imputation-based analysis	
	HR (95% CI)	*P* value	HR (95% CI)	*P* value
**Marital status**				
Married	Reference	**–**	Reference	**–**
Unmarried	1.30 (0.84, 2.05)	0.23	1.30 (1.17, 1.44)	< 0.0001
**Gender**				
Male	Reference	–	Reference	–
Female	1.30 (0.98, 1.75)	0.06	1.33 (1.23, 1.43)	< 0.0001
**Age at diagnosis**				
10–39	1.18 (0.6, 2.18)	0.58	1.15 (0.90, 1.30)	< 0.07
40–49	1.58 (0.93, 2.68)	0.08	1.80 (1.56, 2.04)	0.045
50–59	Reference	–	Reference	–
60–69	1.11 (0.69, 1.64)	0.75	1.11 (0.99, 1.20)	0.052
70–98	1.26 (0.85, 1.85)	0.23	1.25 (1.13, 1.38)	< 0.0001
**Stages**				
Distant	Reference	–	Reference	–
Regional	0.75 (0.55, 1.00)	0.11	0.76 (0.70, 0.83)	< 0.0001
Localized	0.54 (0.32, 0.93)	0.028	0.54 (0.46, 0.62)	< 0.0001
Others	0.38 (0.19, 0.73)	0.004	0.37 (0.31, 0.44)	< 0.0001
**Pathological grading**				
Well differentiated	0.80 (0.34, 1.85)	0.61	0.99 (0.71, 1.09)	0.263
Moderately differentiated	Reference	–	Reference	–
Poorly differentiated	1.77 (1.52, 2.74)	0.009	1.75 (1.57, 1.96)	< 0.0001
Unknown	2.01 (1.33, 3.22)	0.001	2.01 (1.84, 2.39)	< 0.0001
**Morphology**				
Adenocarcinoma (AC), NOS	Reference	–	Reference	–
Mucin-producing adenocarcinoma	1.15 (0.33, 3.9)	0.81	1.11 (0.81, 1.50)	0.499
Mucinous AC	1.31 (0.51, 3.39)	0.09	1.28 (1.00, 1.64)	0.04
Signet ring cell carcinoma	0.91 (0.43, 1.91)	0.79	0.98 (0.81, 1.19)	0.89
AC in villous/tubuvillous adenoma	1.12 (0.32, 3.88)	0.86	1.11 (0.78, 1.49)	0.63
Others	0.61 (0.32, 1.16)	0.13	0.61 (0.53, 0.71)	< 0.0001
**Chemotherapy**				
Yes	Reference		Reference	
No	2.40 (1.59, 3.50)	< 0.0001	2.40 (2.18, 2.66)	< 0.0001
**Surgery**				
Yes	Reference		Reference	
No	1.70 (1.20, 2.40)	0.0007	1.60 (1.52, 1.79)	< 0.0001
**Radiotherapy**				
Yes	Reference		Reference	
No	1.59 (0.93, 2.73)	0.08	1.35 (1.18, 1.55)	< 0.0001

^a^Data represent Saudi patients registered in MNG-HA hospitals system between January 1 2009 and December 31 2017.

^b^Complete cases including missing values.

**Table 3:** Logistic regression analysis for the association of marital status and stage at diagnosis in colorectal cancer patients.

	Stage at diagnosis (advanced vs. localized)^a^	
	OR (95% CI)	*P* value
**Marital status**		
Married	Reference	–
Unmarried	1.52 (1.33, 1.73)	< 0.0001
**Gender**		
Female	Reference	–
Male	0.81 (0.74, 0.87)	< 0.0001
**Age at diagnosis**		
18–39	1.20 (1.01, 1.45)	0.041
40–49	1.36 (1.78, 1.58)	< 0.0001
50–59	Reference	–
60–69	1.10 (0.98, 1.22)	0.09
70–98	0.95 (0.85, 1.11)	0.37
**Pathological grading**		
Well differentiated	1.13 (0.90,1.40)	0.27
Moderately differentiated	Reference	–
Poorly differentiated	2.27 (1.85, 2.79)	< 0.0001
Unknown	0.93 (0.82, 1.10)	0.33
**Morphology**		
Adenocarcinoma (AC), NOS	Reference	–
Mucin-producing AC	1.44 (0.95, 2.17)	0.083
Mucinous AC	1.39 (1.16, 1.67)	0.0003
Signet ring cell carcinoma	2.79 (1.16, 4.82)	0.0002
AC in villous/tubuvillous adenoma	0.38 (0.29, 0.49)	< 0.0001
Others	0.40 (0.33, 0.47)	< 0.0001
**Chemotherapy**		
Yes	Reference	–
No	0.31 (0.28, 0.34)	< 0.0001
**Surgery**		
Yes	Reference	–
No	2.10 (1.88, 2.23)	< 0.0001
**Radiotherapy**		
Yes	Reference	–
No	0.81 (0.69, 0.96)	0.015

^a^Multiple imputation-based analysis.

## Discussion

Survival and other disease-related outcomes in CRC patients have been linked to several factors. However, most studies focused on clinicopathologic characteristics and therapeutic options^32,33^. Social factors have also been linked to disease progression, specifically marital status, in patients with CRC^14,34^. Several studies identified marital status as an independent prognostic factor in colorectal and other cancers, such as pancreatic, breast, prostate, and others^35–39^. There is no literature related to marital status and CRC prognosis in Saudi Arabia. To the best of our knowledge, this study is the first to investigate this proposed relationship.

The impact of marital status on cancer outcomes has been reported in multiple studies with inconsistent results. Aizer et al. reported one of the largest analyses, using the SEER database for the ten leading cancers in the US, including CRC, and noted that unmarried patients have a higher risk of late presentation and cancer-specific mortality^14^. Lai et al. reported that marital status might be significant for certain cancers; however, such association was not shown in a study done by Goodwin et al.^40,41^. In contrast to the literature examining the impact of marital status on different cancer types, our study focused on specific cancer within a particular population not studied before in our region. The survival analysis revealed that married patients with CRC have superior survival compared to unmarried patients and that unmarried patients are at a higher risk of death from CRC. The unmarried patients are also more likely to present with regional or metastatic disease. Although the unmarried patients represented only 11.9% of the sample, it was a heterogeneous population with the majority being young (18–39 years) or old (70–98 years), which might have affected the study results. Young patients with CRC tend to present with more advanced disease^42^, and the young patients treated with standard therapy guidelines do not necessarily derive the same benefit as older age groups^43,44^. Under-treatment due to several factors, such as performance status, comorbidities, and late presentation, may have contributed to the inferior outcomes in older patient populations.

Saudi Arabia does not have a national CRC screening program, and the majority of patients are diagnosed with regional or metastatic disease^45^. The association between an unmarried patient status with an advanced stage at diagnosis should be interpreted with caution, given the relatively small proportion of unmarried patients in our study. Wang et al. reported that more unmarried patients were diagnosed at stage I and II rectal cancer than married patients^17^.

Some limitations should be considered when interpreting the findings of this study. The quality of marriage was not described in our study, as the data were not available in the cancer registry. A poor quality marriage, specifically marital distress, has been implicated in an inferior immune status, which directly affects survival^46^. The database also lacked therapeutic details such as chemotherapeutic regimens, biological agents, therapy duration, radiotherapy type, surgery to primary versus metastatic disease, and other factors affecting survival. In addition, a change in marital status has not been recorded at different time points of the cancer journey. It was collected only at the time of diagnosis, and it is unknown whether changes in marital status affected the colorectal cancer outcomes.

While it is important to note that a causality link between marital status and survival in colorectal cancer was not attempted in this study, our findings that marital status is positively associated with better survival outcomes highlight the importance of marriage as social support in CRC survival outcomes.

## Conclusion

Our findings have implications for future planning of screening and cancer survivorship programs, and more attention should be given to the vulnerable unmarried population. Interventions supporting the early detection of CRC in this population will be beneficial in improving cancer outcomes. Additionally, due to the lower incidence rate of CRC in women than men, there may be some misconceptions about the lower CRC risk in women and delayed diagnosis^47^. Hence, this could be related to the higher stage at diagnosis and lower survival in women reported in this study.

## Supplementary information

Supplementary Informations.

## Data Availability

The data are available from the Oncology Department but restrictions applies to the availability of these data due to sensitive identifier that have been used in this study, which were used under license for the current study, and so are not publicly available.

## Abbreviations

CRC: Colorectal cancer
MNG-HA: Ministry of National Guard-Health Affairs
ICD: International Classification of Diseases
