# Supplementary material for: The effect of marital status on stage at diagnosis and survival in Saudis diagnosed with colorectal cancer: cancer registry analysis
Source: Sci Rep. 2021 Apr 21;11:8603. doi: 10.1038/s41598-021-88042-9 (PMC8060407; doi:10.1038/s41598-021-88042-9)
Supplement: Supplementary file 1 — Supplementary Informations. [file 41598_2021_88042_MOESM1_ESM.docx]

The effect of marital status on stage at diagnosis and survival in Saudis diagnosed with colorectal cancer: Cancer registry analysis

Mesnad Alyabsi, MS, MBA, Ph.D.

Majed Ramadan, PhD

Mohammed Algarni, MD

Kanan Alshammari. MD

Abdul Rahman Jazieh, MD, MPH

| **Supplementary Table S1. The characteristics of the excluded non-Saudi population by nationality** | | | | |
| --- | --- | --- | --- | --- |
| **NATIONALITY** | **Frequency** | **Percent** | **Cumulative Frequency** | **Cumulative Percent** |
| **Afghanistan** | 2 | 0.20 | 2 | 0.20 |
| **Bahrain** | 5 | 0.49 | 7 | 0.69 |
| **Bangladesh** | 1 | 0.10 | 8 | 0.79 |
| **Displaced** | 4 | 0.40 | 12 | 1.19 |
| **Egypt** | 3 | 0.30 | 15 | 1.48 |
| **India** | 2 | 0.20 | 17 | 1.68 |
| **Iraq** | 3 | 0.30 | 20 | 1.98 |
| **Jordan** | 1 | 0.10 | 21 | 2.08 |
| **Kuwait** | 6 | 0.59 | 27 | 2.67 |
| **Malaysia** | 1 | 0.10 | 28 | 2.77 |
| **Palestinian** | 4 | 0.40 | 32 | 3.16 |
| **Pakistan** | 4 | 0.40 | 36 | 3.56 |
| **Philippines** | 9 | 0.89 | 45 | 4.45 |
| **Saudi Arabia** | 936 | 92.49 | 981 | 96.94 |
| **Somalia** | 2 | 0.20 | 983 | 97.13 |
| **South Africa** | 2 | 0.20 | 985 | 97.33 |
| **Sudan** | 8 | 0.79 | 993 | 98.12 |
| **Syrian Arab Republic** | 12 | 1.19 | 1005 | 99.31 |
| **United Kingdom** | 1 | 0.10 | 1006 | 99.41 |
| **United States of Ame** | 1 | 0.10 | 1007 | 99.51 |
| **Unknown** | 2 | 0.20 | 1009 | 99.70 |
| **Yemen** | 3 | 0.30 | 1012 | 100.00 |

| **Supplementary Table S2. Length of follow up among the non-Saudi population** | |
| --- | --- |
| **Length of follow up by year** | **Non-Saudi patients** |
| 0 | 38 (50%) |
| 1 | 16 (21%) |
| 2 | 12 (15.8%) |
| 3 | 3 (3.95%) |
| 4 | 3 (3.95%) |
| 5 | 2 (2.63%) |
| 6 | 2 (2.63%) |
| 7 | 0 |
| 8 | 0 |

| **Supplementary Table S3. Marital Status for non-Saudi patients CRC** | | |
| --- | --- | --- |
| **Married** | **Single** | **Unknown** |
| 42 (55.26%) | 3 (3.95%) | 31 (40.8%) |

| **Supplementary Table S4. The Impact of Marital Status on Colorectal Cancer Risk of Death Stratified by Gender, MNG-HA, 2009-2017.^a^  Multiple imputation-based analysis** | | | | | | | | |
| --- | --- | --- | --- | --- | --- | --- | --- | --- |
|  | **Male** | | | | **Female** | | | |
|  | **HR**  **(95% CI)** | **Pvalue** | **aHR^b^**  **(95% CI)** | **Pvalue** | **HR**  **(95% CI)** | **Pvalue** | **aHR^b^**  **(95% CI)** | **Pvalue** |
| **Marital status** |  |  |  |  |  |  |  |  |
| Married | 1.0 |  | 1.0 |  | 1.0 |  | 1.0 |  |
| Unmarried | 0.72  (0.59,0.88) | 0.001 | 0.73  (0.58,0.91) | 0.007 | 1.29 (1.15,1.44) | <0.001 | 0.96 (0.85,1.09) | 0.55 |
| ^a^ Data represent Saudi patients registered in the MNG-HA hospitals system between January 1, 2009, and December 31, 2017.  ^b^ aHR: Adjusted hazard ratio. Adjusted for all variables in Table 2. | | | | | | | | |
